# Supplementary material for: The Effect of Different Electric Toothbrush Technologies on Interdental Plaque Removal: A Systematic Review with a Meta-Analysis
Source: Healthcare (Basel). 2024 May 16;12(10):1035. doi: 10.3390/healthcare12101035 (PMC11121692; doi:10.3390/healthcare12101035)
Supplement: Supplementary file 1 [file healthcare-12-01035-s001.zip › healthcare-2955386-supplementary.pdf]

__Table S1:__

Cohen's Kappa Agreement Scores
Title screening = 0.94
Abstract screening = 0.89
Full-text screening = 0.92

Excluded studies from manual search

Paper	Reason for exclusion (if applicable)
Williams K, Rapley K, Huan J, Walters P, He T, Grender J, Biesbrock AR. A study comparing the plaque removal efficacy of an advanced rotation-oscillation power toothbrush to a new sonic toothbrush. *The Journal of Clinical Dentistry*. 2008 Jan 1;19(4):154-8.	No interproximal plaque outcome
Williams K, Rapley KA, Haun J, Walters P, He T, Grender J, Biesbrock AR. Comparison of rotation/oscillation and sonic power toothbrushes on plaque and gingivitis for 10 weeks. *American Journal of Dentistry*. 2009 Dec 1;22(6):345.	No interproximal plaque outcome
Goyal CR, Qaqish J, He T, Grender J, Walters P, Biesbrock AR. A randomized 12-week study to compare the gingivitis and plaque reduction benefits of a rotation-oscillation power toothbrush and a sonic power toothbrush. *Journal of Clinical Dentistry*. 2009 Jan 1;20(3):93.	No interproximal plaque outcome
Sharma NC, Qaqish JG, Klukowska MA, Rooney J, Grender JM, Hoke P, Cunningham P. Plaque removal by oscillating-rotating power toothbrush and manual toothbrush. *J Dent Res*. 2010;89.	No sonic toothbrush
Buechel B, Reise M, Klukowska M, Grender J, Timm H, Ccahuana-Vasquez RA, Sigusch BW. A 4-week clinical comparison of an oscillating-rotating power brush versus a marketed sonic brush in reducing dental plaque. *American Journal of Dentistry*. 2014 Feb 1;27(1):56-60.	Sonic toothbrush is multi-directional
